# Surgical and Oncological Outcomes of Minimally Invasive Left Pancreatectomy for Pancreatic Cancer: Robotic vs. Laparoscopic Approach

**DOI:** 10.3390/curroncol32070376

**Published:** 2025-06-28

**Authors:** Matteo De Pastena, Gabriella Lionetto, Salvatore Paiella, Martina Maruccio, Federico Faustini, Elisa Venturini, Antonio Pea, Fabio Casciani, Giuseppe Malleo, Alessandro Esposito

**Affiliations:** 1 Pancreatic Surgery Unit, University of Verona Hospital Trust, 37129 Verona, Italy; martina.maruccio93@gmail.com (M.M.); fede.faustini@gmail.com (F.F.); fabio.casciani@aovr.veneto.it (F.C.); alessandro.esposito@aovr.veneto.it (A.E.); 2 University of Verona, 37129 Verona, Italyelisa.venturini@univr.it (E.V.); antonio.pea@univr.it (A.P.); giuseppe.malleo@univr.it (G.M.); 3 Pancreatic Surgery Unit, Department of Surgery, Dentistry, Paediatrics and Gynaecology, University of Verona, 37129 Verona, Italy; 4 Pancreatic Surgery Unit, Department of Engineering for Innovation Medicine (DIMI), University of Verona, 37129 Verona, Italy

**Keywords:** ductal adenocarcinoma, distal pancreatectomy, robotic distal pancreatectomy, laparoscopic distal pancreatectomy, prognosis, survival

## Abstract

Objective: This study compares the surgical and oncological outcomes of minimally invasive robotic (RLP) and laparoscopic (LLP) left pancreatectomy in pancreatic cancer (PC) patients. Methods: Data from patients who underwent minimally invasive left pancreatectomy between 2013 and 2023 were analyzed. Two groups were identified: RLP and LLP. Perioperative outcomes were compared, including operative time, blood loss, conversion rate, and postoperative complications. Oncological outcomes included margin status, lymph node retrieval, lymph node status, overall survival (OS), and disease-free survival (DFS). Results: Fifty-four patients were divided into the LLP (*n* = 39) and RLP (*n* = 15) groups. The median operative time was shorter for LLP than RLP [260 min vs. 366 min, *p* = 0.007]. Blood loss and conversion rates were comparable (*p* > 0.05). In the LLP group, significantly more lymph nodes were harvested (29 vs. 19, *p* = 0.05), and a higher percentage of positive lymph nodes was noted (72% vs. 40%, *p* = 0.033). No significant difference was found in the R0 resection status (82% vs. 73%, *p* = 0.358). After a median follow-up of 26 months, OS (23 months vs. 34 months, *p* = 0.812) and DFS (17 months vs. 16 months, *p* = 0.635) were similar. Conclusion: RLP provides outcomes identical to LLP in treating body–tail pancreatic cancer, with further studies needed to confirm its long-term oncological efficacy.

## Simple Summary

Minimally invasive left pancreatectomy, including laparoscopic and robotic approaches, has increasingly become the standard for treating pancreatic cancer, particularly for tumors located in the body and tail of the pancreas. Previous research has indicated that both robotic and laparoscopic surgeries offer advantages such as reduced trauma, shorter hospital stays, and faster patient recovery compared to traditional open surgery. However, cancer-related benefits were unclear. Recent studies confirm that both techniques achieve comparable effectiveness in terms of safety, tumor removal success, and patient survival. These results suggest that neither approach is superior in terms of cancer outcomes, highlighting the need for further research. Clarifying these findings through future studies will help inform surgeon training, guide healthcare policy decisions, and optimize surgical strategies, potentially improving patient outcomes and cost-effectiveness in the treatment of pancreatic cancer.

## 1. Introduction

Minimally invasive surgery has dramatically transformed the surgical management of pancreatic diseases, driven by technological advancements and increasing expertise in laparoscopic techniques [1]. Minimally invasive left pancreatectomy (MILP), in particular, has emerged as the preferred treatment approach for resectable pancreatic neoplasms of the body and tail, providing benefits such as reduced surgical trauma, shorter hospitalization, less postoperative pain, and faster patient recovery [2,3,4,5].

Despite these advantages, the optimal minimally invasive approach—robotic left pancreatectomy (RLP) versus laparoscopic left pancreatectomy (LLP)—remains controversial, particularly regarding surgical complexity, oncological outcomes, and cost-effectiveness [6,7,8]. With its high-definition, three-dimensional visualization and enhanced precision, robotic-assisted surgery has gained increasing attention, especially for complex cases involving malignant neoplasms [9,10]. The robotic platform’s ability to offer finer instrument control and improved dexterity may provide an advantage in challenging oncological resections, such as those involving extensive tissue infiltration or desmoplastic reactions, where anatomical planes are difficult to identify. However, evidence comparing these two approaches for treating pancreatic cancer (PC) remains limited and often inconclusive. Previous comparative studies have yielded conflicting results: some suggest superior lymph node retrieval and lower conversion rates with robotic surgery, while others report shorter operative times and fewer complications with laparoscopy [11,12,13]. Recent large-scale studies further underscore these controversies. For instance, Chang et al. reported significantly lower conversion rates to open surgery with robotic procedures, although technical oncological outcomes were comparable between robotic and laparoscopic methods [14]. Another extensive international, multicenter, retrospective cohort study—including 542 patients who underwent MILP for PC—reported higher lymph node yields and lower conversion rates with RLP, but shorter operative times, fewer major complications, and reduced hospital stays with LLP. Regarding oncological outcomes, both techniques demonstrated similar results in terms of radical resection rates and overall survival (OS) [15,16]. Additionally, Ausania et al. highlighted the advantages of robotic pancreatectomy in obese patients, showing lower blood loss, reduced conversion rates, and fewer severe complications compared to laparoscopy [17].

Given these mixed findings, a critical need remains for further comparative research. The present study aims to evaluate and compare perioperative and oncological outcomes between RLP and LLP, addressing current controversies and contributing to the evidence base for the treatment of PC.

## 2. Materials and Methods

This retrospective, single-center study analyzed prospectively collected data from adult patients with a final diagnosis of pancreatic ductal adenocarcinoma (PDAC) who underwent MILP at the General and Pancreatic Surgery Unit of the University of Verona, Italy, between January 2013 and December 2023. The prospective data collection was approved by the local Ethics Committee (PAD-R #1101CESC), and all patients provided informed consent.

The procedures were performed by four different surgeons, all of whom had extensive experience in MI pancreatic resections. Each member of the MI team had achieved proficiency in both laparoscopic and robotic techniques. Given this expertise, surgical board discussions were conducted primarily to confirm the feasibility of a minimally invasive approach for each case. Typically, one robotic and one laparoscopic operating room (OR) are dedicated to left pancreatectomy cases per week, and the type of MI approach is randomly assigned based on the surgical schedule and the availability of the robotic console.

### 2.1. Surgical Procedures

Both procedures have been described in detail elsewhere [18]. During the study period, the technique remained unchanged in terms of procedural steps, the transection technique employed, and the difficulty level.

Briefly, for LLP, the patient is positioned supine at a 20–25° reverse Trendelenburg and a 15–20° right tilt. The open technique was adopted for both procedures, creating a pneumoperitoneum at the umbilicus with a CO_2_ pressure of 12 mmHg. The laparoscopic trocar positions are as follows: the first 12 mm trocar is placed above the umbilicus (camera). Then, a 5 mm trocar is inserted into the epigastrium, positioned beneath the left costal margin. The third 5 mm trocar is located in the right hypochondrium, along the midclavicular line and above the transverse umbilical line. Finally, a 12 mm port is placed in the left hypochondrium, lateral to the umbilicus and along the midclavicular line. An additional 5 mm port may be placed more laterally in the left hypochondrium to optimize exposure. In RLP, robotic docking is performed at the patient’s head, which is in a supine position, at a distance of at least 15 cm from the operating table. Five trocars are used: four 8 mm robotic ports along a transverse umbilical line (R1, in the right flank—R2, in the right pararectal area—R3, in the periumbilical area (camera)—R4, in the left flank), and a 12 mm assistant port underneath and between R3 and R4. Radical antegrade modular pancreatosplenectomy was routinely performed for pancreatic body and tail malignant tumors associated with a standard lymphadenectomy.

The management of the pancreatic stump has been reported previously, involving a stapler reinforced with PGA felt (NEOVEIL^®^ Endo GIA™ Reinforced Reload with Tri-Staple™ Technology 60 mm; COVIDIEN, North Haven, CT, USA) or an ultrasonic dissector (HARMONIC FOCUS or ACE^®^; Johnson & Johnson Medical, Ethicon, Somerville, NJ, USA) [19]. No additional sutures or patches were added during either technique.

Perioperative care followed a standardized enhanced recovery protocol, which included the omission or early removal of drains at the surgeon’s discretion.

### 2.2. Data Collection, Outcomes, and Definitions

Patient data were collected and entered into a secure, prospectively maintained database (REDCap, Vanderbilt University, Nashville, TN, USA). The dataset included demographic variables (age, sex, body mass index (BMI), American Society of Anesthesiologists (ASA) score, diabetes status, neoadjuvant therapy), intraoperative data (operative time including robotic docking, conversion to open surgery, major vessel resection as defined by the ISGPS [20], pancreatic transection method, additional organ resection, estimated blood loss, intraoperative transfusions, drain placement), and postoperative outcomes (complications, length of stay, readmission, reoperation). Pathological data included tumor size, resection margin status, lymphovascular and perineural invasion, and the number of harvested and positive lymph nodes. Oncological outcomes, including the administration of adjuvant therapy, OS, and disease-free survival (DFS), were also assessed. Postoperative complications were classified using internationally recognized grading systems, including the Clavien–Dindo classification for surgical complications [21,22,23,24]. OS was defined as the time from surgery to death or last follow-up, while DFS was defined as the time from surgery to disease recurrence.

### 2.3. Statistical Analysis

Descriptive statistics were utilized to summarize patient characteristics and outcomes. Continuous variables were reported as mean ± standard deviation (SD) or median with interquartile range (IQR), depending on the data distribution. Categorical variables were presented as frequencies and percentages. The Kaplan–Meier method was employed to estimate OS and DFS, and the log-rank test was used to compare survival outcomes between the LLP and RLP groups. Differences in continuous variables were evaluated using the Mann–Whitney U test, while categorical variables were compared with the chi-square test or Fisher’s exact test, as appropriate. A *p*-value of ≤0.05 was considered statistically significant. Statistical analyses were conducted using Stata (version 14.0, StataCorp., College Station, TX, USA) and SPSS (version 25.0, IBM Corp., Armonk, NY, USA).

## 3. Results

A total of 54 patients underwent MILP for PDAC during the study period. Of these, 39 patients (72%) were treated with LLP and 15 (28%) with RLP. No clinically relevant differences were observed in baseline characteristics between the two groups (Table 1).

### 3.1. Intraoperative Data

The median operative time was significantly longer in the RLP group compared to the LLP group (366 min (IQR 275–460) vs. 260 min (IQR 230–312), *p* = 0.007). Major vessel resections, classified as type I ISGPS vein resections, were more frequent in the RLP group (15% vs. 2%, *p* = 0.016). The conversion rate was higher in the LLP group than in the RLP group (8% vs. 0%), although this difference was not statistically significant (*p* = 0.368). Estimated blood loss was similar between the two groups, and there were no significant differences in the management of the pancreatic stump (Table 2).

### 3.2. Postoperative Complications and Outcomes

Postoperative complications occurred in 31% of patients in the LLP group and 40% in the RLP group; however, this difference was not statistically significant (*p* = 0.369). None of the individual complications assessed showed a significant difference between the two approaches (Table 3).

### 3.3. Pathological Data

A significantly higher number of lymph nodes were harvested in the LLP group (median 39 [IQR 28–45] vs. 31 [IQR 29–35], *p* = 0.050), along with a greater proportion of positive lymph nodes (72% vs. 40%, *p* = 0.033). The lymph node ratio (LNR) was also significantly higher in the LLP group [median 0.45 (IQR 0.0–0.12) vs. 0.0 (IQR 0.0–0.04), *p* = 0.016]. Notably, adjuvant therapy was administered more frequently in the LLP group (90% vs. 53%, *p* = 0.016) (Table 4).

### 3.4. Oncological Outcomes

**Figure 1:**
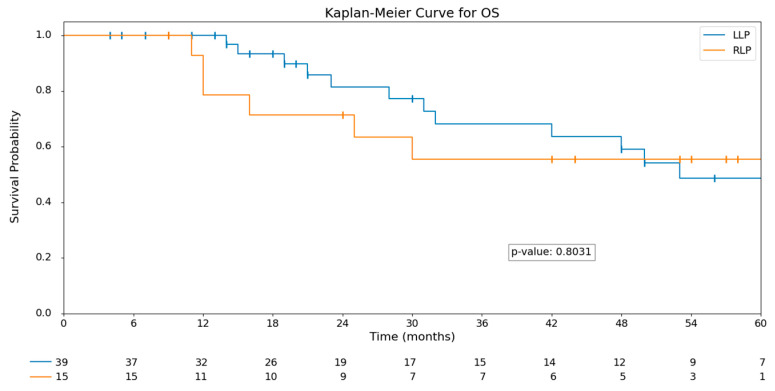
Kaplan-Meier OS analysis: LLP vs. RLP.

**Figure 2:**
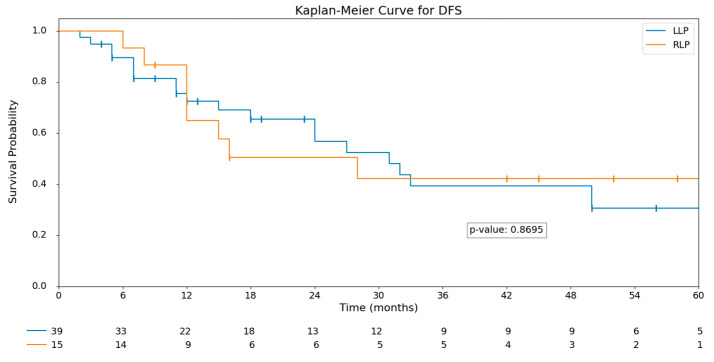
Kaplan-Meier DFS analysis: LLP vs. RLP.

After a median follow-up of 26 months (IQR 14.3–53), no statistically significant differences were observed in recurrence rates between the groups (*p* = 0.603). Similarly, overall survival (OS) and disease-free survival (DFS) showed no significant differences. The median OS in the RLP group was 30 months (IQR 14–53.5), compared to 23 months (IQR 14.5–51.5) in the LLP group (*p* = 0.803). Kaplan–Meier curves for OS and DFS are displayed in Figure 1 and Figure 2.

## 4. Discussion

This study compares the perioperative and oncological outcomes of LLP and RLP for the treatment of PDAC, demonstrating that the two approaches yield broadly equivalent results. These findings align with previous studies, which have also reported minimal differences in safety and efficacy between the two techniques [12,15,25]. The few statistically significant differences observed—such as longer operative times and a higher frequency of major vessel resections in the RLP group—offer valuable insight into the nuanced distinctions between the approaches.

The significantly longer operative time observed in the RLP group is likely attributable to the specific technical demands of robot-assisted surgery, including docking, patient positioning, console setup, and frequent instrument exchanges. Similar findings have been reported by Raoof et al. and Shin et al., who noted extended operative times for robotic procedures but highlighted other benefits, such as reduced conversion rates [12,26]. Our results confirm the trend toward lower conversion rates to open surgery with RLP, in agreement with the previous literature [27]. The enhanced dexterity, instrument stability, and three-dimensional visualization provided by the robotic platform must be weighed against the increased procedural duration. Notably, despite the longer operative times, we found no significant differences in conversion rates, estimated blood loss, or postoperative complication rates, supporting the comparable safety profiles of RLP and LLP.

The safety and precision of the robotic system are further underscored by the significantly higher rate of major vessel resections in the RLP group (15% vs. 2%, *p* = 0.016). This suggests that the robotic approach may better facilitate complex dissections and improve hemostatic control [28,29]. Kauffmann et al. also emphasized the robotic system’s advantages in managing vascular structures, reinforcing its potential role in anatomically challenging oncologic resections.

Interestingly, contrary to prior studies suggesting superior lymph node retrieval with RLP, our study found higher lymph node yields in the LLP group [11,30]. This discrepancy may reflect differences in surgical technique, patient selection, or surgeon experience across institutions. While Chen et al. demonstrated improved lymph node retrieval with RLP [15], Raoof et al. reported no significant difference, highlighting the heterogeneity of results in the literature [26]. Further studies involving larger, more diverse patient cohorts are needed to assess whether our findings are reproducible and clinically meaningful. Nevertheless, both RLP and LLP yielded comparable oncologic outcomes in terms of resection margins, recurrence rates, and overall survival, consistent with existing evidence supporting the oncologic equivalence of these techniques [26,31].

A notable finding was the significantly higher rate of adjuvant therapy administration following LLP (90% vs. 53%, *p* = 0.016). This may reflect differences in patient recovery or case complexity between the groups, with RLP being more frequently used in technically challenging cases that may delay postoperative recovery and, consequently, the initiation of adjuvant therapy [10]. Future studies should evaluate whether such delays impact long-term oncological outcomes.

Overall, our findings support the growing body of evidence indicating that both LLP and RLP are safe and feasible options for the surgical management of pancreatic cancer located in the body or tail of the pancreas. The choice of surgical technique should primarily depend on the surgeon’s proficiency and experience. However, when opting for a robot-assisted approach, longer operative times and higher associated costs should be taken into account [32,33].

This study has several limitations that should be acknowledged. First, its retrospective, non-randomized design inherently introduces the risk of selection bias. Specifically, the choice to perform RLP versus LLP was influenced by factors such as surgical board recommendations, robotic console availability, and possibly the perceived complexity of the case. Consequently, more technically demanding or advanced cases may have been preferentially assigned to the robotic platform, potentially confounding the comparison of perioperative and oncological outcomes between the two groups. For instance, the higher rate of major vessel resections in the RLP group may reflect this underlying selection process rather than an inherent advantage of the robotic approach. Second, variability related to surgeons may have significantly influenced the outcomes. Although all procedures were performed by experienced surgeons skilled in both techniques, the involvement of four different operators introduces heterogeneity in surgical decision-making, technical execution, and case selection. Differences in individual surgeon preferences—such as the extent of lymphadenectomy, the technique for pancreatic transection, or criteria for drain placement—could have contributed to the observed variability in lymph node yields and postoperative management. Additionally, we did not conduct an analysis accounting for the learning curve associated with either approach. This is particularly relevant for robotic surgery, which may have been adopted at different timepoints and with varying caseloads among the surgeons involved. Without controlling for the level of experience at the time of each procedure, it is difficult to isolate the impact of the surgical platform itself from the influence of operator proficiency. Lastly, the relatively small sample size, especially in the RLP group, limits the statistical power to detect small but potentially meaningful differences and restricts the generalizability of the findings to other centers with different patient populations or surgical practices.

## 5. Conclusions

In conclusion, our findings support the equivalence of RLP and LLP regarding perioperative and oncological outcomes in pancreatic cancer treatment. Both approaches are safe and effective, and the surgical choice should consider the surgeon’s expertise, complexity of the procedure, operative time, and cost. Future research, particularly large-scale randomized studies, will help clarify the comparative advantages and long-term outcomes associated with these minimally invasive techniques.

## Figures and Tables

**Table 1:** Demographic data.

Study Population *n* = 54			
Variable, *n* (%)	LLP 39 (72)	RLP 15 (28)	*p*-Value
Age (years, IQR)	65 (59–71)	66 (63–73)	0.258
Sex (Female)	16 (41)	11 (73%)	**0.033**
BMI (kg/m^2^, IQR)	24 (22–27)	24 (22–27)	0.757
ASA score > II	9 (23)	2 (13)	0.350
Diabetes	7 (18)	2 (13)	0.516
Neoadjuvant treatment	6 (15)	5 (33)	0.139

Values in bold are statistically significant. BMI: body mass index; ASA: American Society of Anesthesiology.

**Table 2:** Intraoperative and pathological data.

Study Population *n* = 54			
Variable, *n* (%)	LLP 39 (72)	RLP 15 (28)	*p*-Value
Conversion rate	3 (8)	0 (0)	0.368
Operating time (min, IQR) Major vessel resection	260 (230–312) 1 (2)	366 (275–460) 8 (15)	**0.007** **0.016**
Pancreatic transection technique			0.483
Stapler	33 (85)	12 (80)	
Ultrasonic scalpel	6 (15)	3 (20)	
Additional organs resection	4 (10)	1 (7)	0.572
EBL (cc, median, IQR)	50 (0–275)	50 (0–100)	0.757
Intraoperative blood transfusion	1 (3)	0 (0)	0.722
Drain placement	30 (77)	14 (93)	0.159

Values in bold are statistically significant. IQR: interquartile range; EBL: estimated blood loss.

**Table 3:** Surgical outcomes.

Variable, *n* (%)	Total 54	LLP 39 (72%)	RLP 15 (28%)	*p*-Value
Any complications	18 (31)	12 (31)	6 (40)	0.369
Clinically relevant POPF	7 (10)	4 (10)	3 (20)	0.295
Organ/Space SSI	6 (10)	4 (10)	2 (13)	0.539
DGE	1 (3)	1 (3)	0 (0)	0.722
PPH	5 (10)	4 (10)	1 (7)	0.572
Pneumonia	4 (8)	3 (8)	1 (7)	0.694
Cardiovascular complications	5 (10)	4 (10)	1 (7)	0.572
Clavien–Dindo score ≥ 3	4 (10)	4 (10)	0 (0)	0.260
Length of stay (days, median, IQR)	7 (6–9)	7 (6–9)	7 (6–9)	0.444
Reoperation	2 (5)	2 (5)	0 (0)	0.518
Readmission	2 (5)	2 (5)	0 (0)	0.518

POPF: postoperative pancreatic fistula; DGE: delayed gastric emptying; PPH: post-pancreatectomy hemorrhage.

**Table 4:** Pathological data.

Study Population *n* = 54			
Variable, *n* (%)	LLP 39 (72)	RLP 15 (28)	*p*-Value
Tumor size (mm, median, IQR)	27 (21–35)	27 (18–35)	0.720
R0 margin	32 (82)	11 (73)	0.358
Lymphovascular invasion	33 (85)	11 (73)	0.278
Perineural invasion	35 (90)	12 (80)	0.295
Harvested lymph nodes (median, IQR)	39 (28–45)	31 (29–35)	**0.050**
Positive lymph nodes	28 (72)	6 (40)	**0.033**
Lymph node ratio (median, IQR)	0.45 (0.0–0.12)	0.0 (0.0–0.04)	**0.016**
Adjuvant therapy	34 (90)	8 (53)	**0.016**

Values in bold are statistically significant. IQR: interquartile range; EBL: estimated blood loss.

## Data Availability

The original contributions presented in this study are included in the article. Further inquiries can be directed to the corresponding author.

## Abbreviations

The following abbreviations are used in this manuscript:

RLP	Robotic left pancreatectomy
LLP	Laparoscopic left pancreatectomy
PC	Pancreatic cancer
OS	Overall survival
DFS	Disease-free survival
MILP	Minimally invasive left pancreatectomy
